# Inclusion of older adults in UK clinical research—the National Institute for Health and Care Research Year of Birth Project

**DOI:** 10.1093/ageing/afag143

**Published:** 2026-05-27

**Authors:** Martyn Patel, Divya Tiwari, Shamaila Anwar, Philip Evans, Chloe Meersschaert, Jatinder S Minhas, Jane A H Masoli, Terence J Quinn

**Affiliations:** Norfolk and Norwich University Hospitals NHS Foundation Trust, Older Peoples Medicine, 7 Colney Lane, Norwich NR4 7UY, UK; University Hospitals Dorset NHS Foundation Trust, Poole, Bournemouth, Christchurch, UK; NIHR—RDN Coordinating Centre, Leeds, UK; University of Exeter, Medical School (Primary Care), Exeter, UK; Universite de Poitiers, Faculte de Sciences, Poitiers, Nouvelle-Aquitaine, France; University of Leicester, Division of Cardiovascular Sciences, Leicester, UK; University of Exeter, Epidemiology and Public Health, University of Exeter Medical School, College House, University of Exeter Medical School, St Luke's Campus Heavitree Road, Exeter EX4 4QJ, UK; Royal Devon and Exeter NHS Foundation Trust, Healthcare for Older Adults, Barrack Road, Exeter EX2 5DW, UK; University of Glasgow, Cardiovascular and Medical Sciences, Glasgow Royal Infirmary, Walton Building, Glasgow G4 0SF, UK

**Keywords:** inclusion, older adults, research, trials

## Abstract

**Introduction:**

Inclusion of older adults in research ensures both equity and relevance. We used National Institute of Health and Care Research Year of Birth data to describe inclusion of older adults in contemporary UK clinical research.

**Methods:**

We worked with English research networks to collect individual participant level data on recruits into National Institute of Health and Care Research portfolio studies. We used data from 2022–2023 to 2023–2024 to assess participation. We described data in terms of geography, primary speciality managing the study and study characteristics. In subgroup analyses, we assessed inclusion of the ‘oldest old’ (aged over 85 years) and inclusion in research from three exemplars: heart failure, Parkinson’s disease and palliative care.

**Results:**

In total, 455 734 participants were included in the analyses. The most common age group included in UK research was 65–74 years (15.5%). People aged over 85 years were 3.2% of all recruits, and this proportion decreased over time. There was modest geographical variation in participant age but marked variation by recruiting speciality. People aged over 85 years accounted for less than 5% of the total participants recruited in 9 of 14 specialities assessed. Older adults were less likely to be recruited into commercial studies. For three conditions of interest, recruitment did not match population epidemiology.

**Conclusion:**

This project has demonstrated feasibility of collecting participant age data at scale and provides important metrics for understanding study participation. While there is scope to improve older adult participation across all the study portfolio, there are major equity issues around inclusion in certain speciality areas and commercial research. *To complement this abstract, a video abstract is available online*.

## Key points

- It is feasible to collect data on the age of clinical research participants at national scale.
- Inclusion of older adults (aged over 85 years) is uncommon, and decreasing, in UK clinical research.
- There is variation in the inclusion of older adults by geography, study sponsor, and clinical speciality.
- The National Institute of Health and Care Research Year of Birth data can be used to track progress against the national priority of increasing the numbers of older adults participating in clinical research.

## Introduction

For a clinical study to have real-world relevance, the profile of the study population needs to be as close as possible to the intended patient population. However, this is often not the case [1, 2]. There are many instances of treatments, studied almost exclusively in one subset of the population, that are later shown not to have equivalent effectiveness in other, sometimes larger, patient groups. Examples include ethnic difference in ischaemic heart disease prevention and blood pressure control strategies [3, 4].

A group that has been historically under-represented in healthcare research are older adults [5]. This means that geriatric medicine as a speciality has one of the worst ratios of clinical trial evidence to patient numbers. This evidence-practice disconnect is a substantial missed opportunity. The majority of the hospital inpatient population, and a high proportion of health and social care expenditure are related to older adults [6]. At worst, this represents inherent ageism in clinical research and will lead to clinical practice recommendations that are not relevant to the main consumers of health care [7].

It is recognised that recruiting older adults, especially older adults living with frailty, to clinical research studies is challenging. There are many potential barriers to inclusion of this group, including, but not limited to, physical and cognitive impairments, multiple long-term conditions and reduced healthcare literacy. These practical difficulties of recruitment can be compounded by study protocols that set arbitrary upper age thresholds for inclusion, or stipulate other criteria that will exclude the majority of older adults [8].

However, recruitment of older adults into multi-centre clinical trials is possible, with recent high profile examples [9, 10], and supporting guidance from international societies [11]. The landscape is changing, and in the UK, best practice around the inclusion of older adults in research has been published [12], the Chief Medical Officer has issued an annual report themed around older adults with recommendations for greater research inclusion [13], and a recent ‘statement of intent’ signed by major UK research funders has underlined a commitment to increasing older adult participation in research [14].

To fully understand the inclusion of older adults in research, to allow us to audit practice and to monitor temporal trends, we need access to timely, robust, data on the ages of research participants at national scale. While most studies report average age as part of baseline demographics, more granular data on the ages of participants are often lacking [15]. In the UK, a large proportion of clinical research studies are eligible to register with the National Institutes of Health and Care Research (NIHR) Research Delivery Network (RDN) (formerly the Clinical Research Network (CRN)) portfolio service [16]. NIHR portfolio inclusion brings many benefits to investigators, but also comes with a requirement for mandatory reporting at the level of the person recruited. Although numbers of participants recruited is routinely reported by local sites via the RDN Central Portfolio Management System, no national data on participant characteristics was traditionally collated. To improve inclusivity and equity in research participation, in 2021 a system to allow routine capture of research participants’ year of birth (YoB) was introduced, as a first step towards building a national dataset on research demographics.

Here, we present initial data from this NIHR’s YoB project, exploring the data through the lens of inclusion of older adults. Our specific aims were to describe patterns of age for participants in UK clinical research by discipline speciality, by geography and by study type.

## Material and methods

### Data collection

Full details of the methods underpinning the NIHR YoB project have been described previously [17]. In brief, the original NIHR YoB pilot was designed to assess the feasibility of collecting participants’ age through existing local systems and to use these data to understand the scale, variation and challenges of individual participant data collection across England. From 2021, the NIHR CRN Coordinating Centre collaborated with the 15 Local Clinical Research Networks (LCRNs) in England to implement data collection. NHS Trusts were asked to report the YoB of participants via their Local Portfolio Management Systems.

### Data selection and categorisation

We limited analyses to data from years 2022–2023 to 2023–2024. Choosing these as years with most complete data availability, and less likely to be impacted by COVID-19 healthcare restrictions.

Deriving age from YoB, we linked these data to other data routinely captured as part of national mandatory reporting. We created classifications to divide the data according to key variables of interest.

#### Age ranges

We pre-specified age ranges of greater than 65 years (an age commonly used to define eligibility for retirement) and age greater than 85 years (an age commonly considered as ‘oldest old’), with a middle threshold of 75 years (recognising the potential heterogeneity in the group 65–85 years old) [18].

#### Clinical speciality

We used the speciality definitions of the NIHR CRN, including the speciality of ‘ageing’, but excluding ‘children’ and ‘reproductive health’. We noted where a speciality ‘managed’ a portfolio study, i.e. were primarily responsible for the successful delivery of the study; and where a speciality ‘supported’ a study, i.e. assisted another speciality who were managing the study.

#### Geography

We used the 15 regions of England that defined the NIHR LCRNs.

#### Study type

We categorised research as clinical trials of an investigational medicinal product (CTIMP i.e. a ‘drug trial’) and non-CTIMP. We also categorised as ‘commercial’ (where a company was the primary sponsor) and ‘non-commercial’ (where a hospital, university or individual investigator was the sponsor).

#### Exemplar conditions

We choose two conditions, and one healthcare context, where relevant patients were likely to be older adults, but that did not traditionally fall under the NIHR CRN ‘ageing’ classification, these were: heart failure, Parkinson’s disease and palliative care studies. These conditions were defined using ICD 10 diagnostic codes and speciality codes. We compared patterns of recruitment with UK data on population prevalence by age [19–21].

### Analyses

We used descriptive statistics, reporting absolute numbers and proportions for our three chosen age bands and by classifications outlined above. Where appropriate, means and proportions were assessed using relevant comparative analyses. We created tables and data visualisations. All descriptive analyses were performed using *IBM SPSS* version 29.0.1.0 software. We created a map comparing proportions of older adults in research and proportions of older adults in the region, using UK census data. This work was fully supported by the NIHR, with no external support. Data were fully anonymous and no additional ethical approvals were required for these analyses.

## Results

In the 4 years since inception, the NIHR YoB project has collected age data for over 1 million participants, with numbers increasing. These people were recruited to over 4900 portfolio studies, across 204 NHS Trusts in England. The average age of NIHR portfolio study participants was 48.4 years, with the most common 10-year age band being those aged 65–74 years, accounting for 15.5% of total UK research participants.

Not all portfolio studies or regions consistently reported YoB data and regional responses varied across the study period. Although overall, the number of studies reporting YOB data increased across the two years from 7343 to 8171 studies.

During the period 2022–2023, 207 490 participants were included in research reporting YoB data, of which 85 945 (41%) were aged over 65 years and 10 726 (5%) aged over 85 years. Equivalent figures for 2023–2024 were 248 244 participants, 95 399 (38%) over 65 years old and 11 401 (4.5%) over 85 years old, a decrease in older adult recruitment (*P* < .0001). Across the relevant portfolio, 4145/5608 (74%) of studies recruited any participants aged over 65 years.

Table 1 describes recruitment by selected NIHR CRN/RDN speciality group for each of our pre-specified age bands. Compared to other RDN/CRN disciplines, recruitment to those studies managed by the ageing speciality was modest. Other than for those studies primarily managed by the ageing speciality group, there was a general pattern of lower proportional recruitment with increasing age. As a proportion of total recruitment, recruitment of the ‘oldest old’ (those aged over 85 years) was single figures for all specialities except ageing, respiratory, stroke and trauma. Low proportional recruitment of this age group was seen for many specialties where older adults would be a major service user group, e.g. cancer, dementia and mental health.

**Table 1:** Age based recruitment by study managing speciality.

Speciality	No. of studies with relevant data/no. of studies 4145/5608	2022–2023 (number and proportion of total recruitment)			2023–2034 (number and proportion of total recruitment)		
		65–75	76–85	>85	65–75	76–85	>85
Ageing	20/21 19/20	166 23.9%	259 37.3%	158 22.7%	287 14.6%	753 38.5%	783 40%
Anaesthesia pain and perioperative	30/43 35/45	3019 27.5%	2324 21.2%	526 4.8%	4240 24%	2747 15.5%	476 2.6%
Cancer	598/757 641/787	7663 27.2%	3767 13.3%	572 2%	10,618 53.2%	4716 23.6%	627 3.1%
Cardiovascular	197/227 205/238	5132 29.4%	4275 24.5%	1093 6.2%	3907 26.2%	3861 25.7%	1128 7.5%
Dementia and neurodegeneration	132/120 140/160	1582 30.7%	1222 23.7%	329 6.3%	1876 31.3%	1637 32,3%	298 4.9%
Diabetes	43/64 49/73	1065 17.3%	515 8.4%	79 1.2%	1073 18.4%	527 9%	94 1.6%
Gastroenterology	81/109 75/104	3824 19.6%	1902 12.6%	217 1.4%	3996 22.1%	2086 11.5%	228 1.2%
Infection	58/104 56/103	1590 14.9%	481 4.5%	209 1.9%	1228 17.5%	551 7.8%	185 2.6%
Mental health	35/131 48/146	184 4.1%	66 1.4%	13 0.2%	416 3.7%	146 1.3%	32 0.2%
Musculoskeletal and orthopaedics	129/178 144/191	2505 24.1%	1357 13%	225 2.1%	2929 22.7%	1614 12.5%	217 1.7%
Neurology	55/97 55/101	2347 19.5%	1674 13.9%	456 3.7%	1519 17.1%	1237 13.9%	327 3.6%
Respiratory	112/134 117/144	4345 22.1%	4188 21.3%	3071 15.6%	4615 23.9%	4378 22.7%	2670 13.8%
Stroke	53/58 60/64	894 23.2%	1068 27.8%	476 12.4%	1022 22%	1254 27%	809 17.4%
Trauma and emergency care	64/76 52/62	2160 15.3%	2395 16.9%	1648 11.6%	2427 17.6%	2490 18.1%	1454 9.8%

Table contains a selection of the NIHR clinical research network specialities.

**Figure 1:**
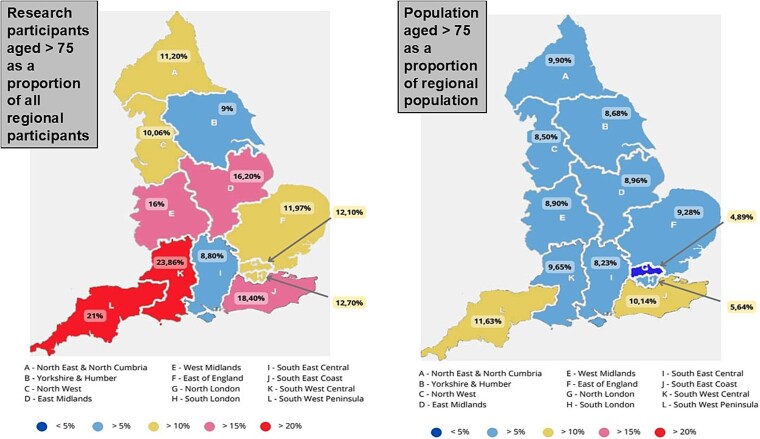
Research participants aged over 75 years as a proportion of all research participants by region, people aged over 75 as a proportion of the regional population. The regions are presented as NIHR Research Delivery Network Regions.

Table 2 presents recruitment by age band, categorised by geography, using the NIHR clinical research network sites, Figure 1 compares research participation by age in RDN regions. There is variation in recruitment of older adults by region. There was no clear relationship between overall research study activity (number of portfolio studies in the region) and inclusion of older adults.

**Table 2:** Age-based recruitment across NIHR clinical research network regions in England.

Region	No. of studies with relevant data/no. of studies	2022–2023 (number and proportion of total recruitment)			2023–2024 (number and proportion of total recruitment)		
		65–75	76–85	>85	65–75	76–85	>85
East Midland	179/255 187/272	1314 (15.8%)	894 (10.5%)	233 (2.7%)	1724 (13.6%)	1370 (10.8%)	684 (5.4%)
East of England	240/328 228/314	2112 (17.7%)	1636 (13.7%)	383 (3.2%)	1863 (13.8%)	1516 (11.2%)	371 (2.7%)
Greater Manchester	97/143 331/522	1569 (17.4%)	1091 (12.1%)	226 (2.5%)	5397 (15.9%)	2542 (7.5%)	707 (2%)
Kent Surrey and Sussex	190/281 198/294	1979 (16.6%)	1877 (15.7%)	753 (6.3%)	2127 (15.3%)	2046 (14.7%)	520 (3.7%)
North East and North Cumbria	480/752 477/734	4824 (14.7%)	2880 (8.7%)	797 (2.4%)	4883 (12.8%)	3269 (8.5%)	1034 (2.7%)
North Thames	301/517 426/698	2861 (19.3%)	2190 (14.7%)	709 (4.7%)	3885 (12.4%)	2802 (8.9%)	679 (2.1)
North West Coast	337/543 347/559	3011 (15.3%)	1971 (10%)	459 (2.2%)	3159 (13.3%)	2061 (8.6%)	495 (2%)
North West London	254/437 270/475	1442 (9.5%)	928 (6.1%)	355 (2.3%)	1718 (12.9%)	1150 (8.6%)	475 (3.5%)
South London	400/670 497/823	2216 (18.8%)	1408 (11.9%)	298 (2.5%)	2815 (15.1%)	1839 (9.8%)	556 (2.9%)
South West Peninsula	346/465 353/459	2641 (20.4%)	1912 (14.8%)	428 (3.2%)	2343 (23.5%)	1710 (17.1%)	388 (3.9%)
Thames Valley and South Midlands	266/422 324/496	1461 (8.3%)	1033 (5.8%)	288 (1.6%)	1902 (7.5%)	1666 (6.6%)	565 (2.2%)
West of England	271/430 278/440	4713 (17%)	4585 (16.5%)	3278 (11.8%)	4328 (17.3%)	4101 (16.3%)	2660 (10.6%)
Wessex	352/528 325/496	3651 (21.5%)	2700 (15.9%)	811 (4.7%)	3052 (20.2%)	2246 (14.9%)	566 (3.7%)
West Midlands	443/756 443/739	3789 (17.5%)	2502 (11.5%)	672 (3.1%)	3444 (15.7%)	2778 (12.7%)	722 (3.3%)
Yorkshire and Humber	513/816 558/850	6496 (16.2%)	3609 (9%)	1065 (2.6%)	6419 (11.7%)	3975 (7.2%)	996 (1.8%)

Table 3 presents recruitment by age band, categorised by study type. Overall, 3579 CTIMP and 2744 non-CTIMP studies reported YOB data in our period of interest. There was no difference in proportions of trials recruiting older adults. However, comparing commercial and non-commercial studies, those aged >85 years were better represented in non-commercial trials to commercial trials (3.5% vs. 2.4%, *P* < .001).

**Table 3:** Age-based recruitment by study type.

Study type 9911/15514	No. of studies with relevant data/no. of studies	2022–2023 (number and proportion of total recruitment)			2023–2034 m (number and proportion of total recruitment)		
		65–75	76–85	>85	65–75	76–85	>85
ALL portfolio	4669/7343 5242/8171	44,079 (15.8%)	31,216 (11.2%)	10,755 (3.8%)	49,059 (14%)	35,071 (10%)	11,418 (3.2%)
Ageing managed	20/4669 21/5242	166 (23.9%)	259 (37.3%)	158 (22.7%)	347 (17.8%)	792 (38.4%)	790 (38.3%)
Ageing supported	56/4669 60/5242	909 (24.5%)	1409 (37.9%)	666 (17.9%)	1237 (34.5%)	1105 (30.8%)	562 (915.6%)
CTIMP	1122/1748 1200/1831	17,372 (16.3%)	11,504 (10.3%)	3554 (3.3%)	19,936 (14.4%)	15,201 (10.9%)	5360 (3.8%)
Non CTIMP	920/1359 939/1385	26,707 (15.5%)	19,712 (11.5%)	7201 (4.2%)	29,123 (13.7%)	19,870 (9.3%)	6058 (2.8%)
Commercial	525/864 603/940	3364 (21.1%)	1868 (11.7%)	400 (2.5%)	3526 (19.6%)	2238 (12.4%)	431 (2.4%)
Non-commercial	1517/2243 1536/2276	40,715 (15.5%)	29,348 (7.7%)	10,355 (3.9%)	45,533 (13.6%)	32,833 (9.8%)	10,987 (3.3%)

CTIMP, Clinical Trial of an Investigational Medicinal Product.

Table 4 presents recruitment by age band for three exemplar conditions and highlights age related decreases in study participation and an overall disproportionately small representation of older adults compared to the community epidemiology of the chosen conditions.

**Table 4:** Age-based recruitment for three exemplar conditions of older age.

Disease area	Managed speciality	No. of studies with data/no. of studies	2022–2023 (number and proportion of total recruitment)			2023–2034 (number and proportion of total recruitment)		
			65–75	76–85	>85	65–75	76–85	>85
Heart failure	UK prevalence		2.9%	6.7%	14.3%	2.9%	6.7%	14.3%
	CV	69/74 71/80	1191 (28.2%)	871 (20.6%)	179 (4.2%)	1257 (27.5%)	1021 (22.3%)	256 (5.6%)
	Other	9/12 8/9	60 (18.9%)	53 (16.7%)	19 (6%)	84 (26.2%)	42 (13.1%)	11 (3.4%)
Parkinson’s	UK prevalence		1.0%	1.4%	1.6%	1.0%	1.4%	1.6%
	Neuro	45/45 46/49	832 (44.3%)	324 (17.2%)	33 (1.7%)	908 (37.3%)	542 (22.2%)	88 (3.6%)
	Other	7/7 11/11	208 (20%)	153 (14.7%)	25 (2.4%)	295 (16.5%)	160 (8.9%)	24 (1.3%)
Palliative care	Median age of death in UK palliative care: 74 years							
	Palliative	19/22 19/22	319 (26.9%)	237 (20%)	54 (4.5%)	510 (30%)	413 (24.3%)	112 (6.5%)
	Other	4/9 4/8	46 (13.1%)	44 (12.6%)	18 (5.1%)	72 (12.5%)	47 (8.1%)	4 (0.6%)

## Discussion

Improving the inclusion of older adults in clinical research is recognised as a priority in the UK [13]. The NIHR YoB project demonstrates encouraging recruitment of those over retirement age, but there is still considerable work to be done, as recruitment of the oldest old remains only a fraction of total UK research activity. Concerningly, as a proportion of total recruitment, older adult participation is decreasing over time, and recruitment patterns are not always representative of the real-world distribution of disease.

Comparing study recruitment to UK census data helps contextualise the YoB data. In the last census, people aged over 65 comprised 19% of the UK population, while those aged over 85 years comprised 2.4% [22]. These percentages compare favourably to the total proportional recruits by age band. However, if we consider the UK population with medical conditions requiring intervention (the primary focus of clinical research), this population are predominantly older adults, confirming the need to improve participation of this group [23]. To assess research participation age against population age, we choose three exemplar conditions, namely heart failure, Parkinson’s disease and palliative care. For heart failure, the majority of UK disease is in those aged over 75 years, with mean age of diagnosis 79 for women [19]. However, in the YoB data, participants recruited were much younger. Similar disconnects between recruitment and UK prevalence patterns were seen for Parkinson’s disease (where peak diagnosis is in those aged over 80 years) [20] and for people requiring palliative care (where the median age of requiring this input is 75 years) [21].

A recognition of the under inclusion of older adults in clinical research is not new. A 2020 paper used condition-specific estimates of research recruitment in diabetes, cardiology and cancer studies to illustrate the paucity of older adults in research [24]. That review noted that a major diabetes trial that informed guidelines had an average age of only 53 years, a meta-review of oncology studies showed less than a third of eligible older adults were recruited to trials [25] and less than a quarter of older Medicare beneficiaries with heart failure met enrolment criteria landmark heart failure studies [26]. While there has been progress made, e.g. the NIHR INCLUDE guidance [12], there is clearly still substantial work to be done. Internationally, this feeling of a journey started, but far from completed, is recognised by those working to improve older adults' inclusion in US research [27].

Increasing older adult participation in research requires not only an increase in studies with a focus on syndromes of older age, but also increasing older adult participation across all studies. Older adults are commonly affected by a variety of organ specific conditions, and if recruitment to studies mirrored the natural epidemiology of these conditions, we would not see patterns of low older adult participation in research with a focus on cancer, dementia and neurodegenerative diseases. Exploring the variation in inclusion of older adults can suggest targets for intervention, e.g. working with commercial sponsors to increase proportions of the oldest old in their trials or selecting conditions where inclusion of this demographic is lower than expected. Specific disease areas such as neurology and cancer have described challenges and solutions to improving research inclusion [28, 29]. The progress made in these areas gives us encouragement to work harder on improving access to research for older adults.

Our work complements a recent project completed by the UK research funder The Vivensa Foundation [30]. They described the UK Ageing research ecosystem and reported that most UK ageing research was funded by public sector (e.g. NIHR). The report recognised age-based disparities beyond inclusion, with research need (availability of evidence versus associated disability adjusted life years) not matching available funding, and majority of ageing-related research being concentrated in London and the South-East England. While these are areas of world leading academic activity, they are not the areas of the UK with the highest prevalence of older adults or frailty. In our analysis comparing regional population ages with ages of those included in research, there is marked regional heterogeneity. Those areas with the largest number of older people, have the highest recruitment of older adults into research, but the regional differences are modest, and it would be wrong to state that UK NIHR study participation perfectly matches population ageing patterns.

Accurate, real-time data on characteristics of participants in research offer a powerful resource to ensure the community are working towards equity and external validity of clinical research. The granularity afforded by individual participant-level data allow for assessment of temporal trends, subgroups and comparisons across regions or disciplines. Continued reporting of YoB will provide a benchmark to measure progress in older adult inclusion at local, national and international level—and may encourage other countries to report similar data.

The YoB data have potential applications from local audit through to guiding national policy. Mapping regional variation in inclusion of older adults, allows both peer-to-peer geographical comparisons, and comparisons within a region, checking recruitment against regional demographics. Certain UK research regions have higher proportions of older adult residents, and we aware that Ageing specialty teams within some of these regions are starting to use the YoB data to guide local research recruitment decisions. At a national level, the data permit UK policy makers to closely examine the impact of interventions such as themed calls for older adult research, and the promotion of inclusion-based study paradigms.

The YoB data offer granular information on age of research participants from across the UK. Despite its many opportunities, there are limitations to the current data. Those working in older adult research will be aware that chronological age is a blunt tool for describing biological age, or the common syndromes associated with older age such as frailty [31]. However, as an objective, easy to collect variable, chronological age may serve as a suitable proxy for use at scale.

Missing data are always an issue with data collection at national scale, and contributing data to the YoB project was not mandatory for trusts or studies. There is potential for biases in the missing data, e.g. those with better inclusion of older adults may be more likely to submit data, and as data are predominantly from secondary care, may not be generalisable to other settings e.g. care-homes or primary care. However, it seems unlikely that the non-participating sites and studies have substantially better inclusion than those that submitted data.

The YoB project has demonstrated feasibility of collecting participant age data at scale across the NIHR portfolio and aids our understanding of who takes part in research and where gaps remain. However, to fully assess equity, other demographic data (such as ethnicity, gender and socioeconomic status) must also be monitored. In this regard, the UK Department of Health and Social Care is piloting a scheme to collate more granular demographic information of research participants, including sex and socioeconomic deprivation. Specific to older adult research, there would be a desire to complement the protected characteristic of age, with data on frailty, and multiple long-term conditions. Achieving this will require negotiating issues around data governance and inter-operability of data sources. These developments in data monitoring are essential if we wish to realise the ambition of making research more inclusive, representative and aligned to population health needs.

‘If you cannot measure it, you can not improve it’. To have reached, a point at which YoB recording is encouraged for all studies on the NIHR portfolio is a major milestone towards a goal of improving the inclusion of older adults in research. The current YoB data show the importance of older adults in UK clinical research but also highlights variation and opportunities to improve participation in particular specialities, localities and study types.

## Data Availability

At present, the individual participant data cannot be shared outside of the NIHR.
